# Evaluation of Seroprevalence and Risk Factors of Heartworm Infection for Dogs in Rio de Janeiro with Access to Veterinary Care

**DOI:** 10.1007/s11686-024-00859-2

**Published:** 2024-07-02

**Authors:** Mariana Guedes, Tamiris Gomes, Bruno Alberigi, Emilene Prudente, Alexandre Bendas, Thalita Souza, Flavya Mendes-de-Almeida, Fabiana Batalha Knackfuss, Alexandre Merlo, Norma Labarthe

**Affiliations:** 1 Zoetis Indústria de Produtos Veterinários Ltda, Rua Dr. Chucri Zaidan, 1240, 4º andar, São Paulo, SP Brazil CEP: 04711-130; 2 Universidade Federal Rural do Rio de Janeiro, Rodovia BR 465, Km 07, Zona Rural, Seropédica, RJ Brazil CEP: 23890-000; 3 Universidade Federal Fluminense, Avenida Ary Parreiras, 507, Icaraí, Niterói, RJ Brazil CEP: 24220-000; 4 Fundação Oswaldo Cruz., Rua Leopoldo Bulhões, 1480 - Manguinhos, Rio de Janeiro, RJ Brazil CEP: 21041-210

**Keywords:** Epidemiology, Canine health, Prevalence, Parasitology, Preventive measures, Clinical signs, Vector-borne diseases, Mosquito vectors, *Dirofilaria immitis*

## Abstract

Heartworm infection is a chronic disease with clinical signs and effects ranging from an asymptomatic condition to severe disease and death. The prevalence of heartworm disease in the state of Rio de Janeiro has been reported to be high (21.3%). The present study was conducted to evaluate the seroprevalence and risk factors of heartworm infection for the canine population with access to veterinary services in different areas of the state of Rio de Janeiro, Brazil. A total of 1787 canine blood samples were obtained from 135 practices across 8 different areas of Rio de Janeiro state (Rio de Janeiro municipality, São Gonçalo municipality, Niterói municipality, Baixada Fluminense, and the northern, southern, eastern, and mountainous areas) and tested for the presence of *Dirofilaria immitis* antigens and antibodies against several tick-borne disease pathogens using a commercial immunochromatography technique (Vetscan® Flex 4 Rapid Test; Zoetis; NJ USA). Pet owners reported living conditions, physical characteristics, demographics, and clinical signs for evaluation of risk factors for heartworm infection. Only two evaluated risk factors were shown to enhance the risk for *D. immitis* infection, including having a short hair coat vs. having a medium or long hair coat (OR 2.62) or positive for antibodies to tick-borne disease parasites (OR 3.83). Clinical signs reported for dogs with heartworm disease were typical for that condition. The overall prevalence of heartworm disease in the state was 8.2%, ranging from 2.4% in the mountainous region to 29.4% in the eastern area. It could not be determined if veterinarians were not diligent about dispensing heartworm preventatives or if poor levels of compliance by dog owners were responsible for higher infection rates in some areas of the state.

## Introduction

Heartworm disease is a cardiopulmonary vector-borne disease caused by *Dirofilaria immitis* and its endosymbiont *Wolbachia*. This nematode infects domestic and wild canids, which are the best-adapted hosts and the main source of microfilariae for mosquito vectors [1]. Although canids are the most infected mammals, many other groups may be infected, including humans [2].

Heartworm disease is a chronic disease that causes vascular damage in pulmonary arteries. Clinical signs are variable and range from an asymptomatic condition to weight loss, decreased exercise tolerance, cough, dyspnea, syncope, ascites, cachexia, caudal vena cava syndrome, and death, which is typically due to pulmonary hypertension and right heart failure [3, 4].

*D. immitis* is a cosmopolitan parasite. The prevalence of canine infections tends to be higher in coastal tropical areas due to conditions that provide abundant, flourishing populations of the vector [5]. In Brazil, canine heartworm disease has been reported in all 5 regions of the country [6, 7]. Besides the vector density, which tends to follow naturally conserved areas, the unprotected canine population, especially stray, feral, or sylvatic animals, increases the risk for new infections [8].

In the state of Rio de Janeiro, the prevalence has been reported to be high (21.3%) [9]. The coastal areas have always been recognized as having the highest number of heartworm cases in the state [10]. After chemoprophylaxis became available in Brazil in the 1990s, infections became rare in Rio de Janeiro, reaching a low level of 3.8%, even when antigen testing was used in former endemic areas [11]. Twenty-four years after the first launch of a macrocyclic lactone, the prevalence started to rebound at previous foci sites, reaching 23.1% in 2014, especially in the coastal area of Rio de Janeiro [12]. For instance, in the eastern region of the state, positive antigen frequencies were reported to be 44.8% [12].

Therefore, since many different factors may influence the environment and mosquito and canine populations, constant surveillance is needed. The present study was conducted to evaluate the seroprevalence and risk factors of heartworm infection for the canine population with access to veterinary services in the state of Rio de Janeiro, Brazil.

## Materials and Methods

### Sampling Criteria

The state was divided into 8 geographical areas (Rio de Janeiro municipality, São Gonçalo municipality, Niterói municipality, Baixada Fluminense, and the northern, southern, eastern, and mountainous areas of the state) based on population size and environmental characteristics.

The number of dogs to be included in each area was calculated with the aid of EPI INFO™ 2000, considering the canine population to be 10% of the human population in each area [13]. The expected canine *D. immitis* infection frequency ranged from 3% in the mountainous region to 30% in the eastern area [12, 14], with a confidence interval (CI) of at least 95%. Therefore, the estimated number of dogs to be enrolled across the state to provide a statistically relevant sample was 1665. The study was carried out from June to December 2021, despite the strict control of human activities in most Brazilian cities due to the COVID-19 pandemic.

The veterinary clinics and practices invited to participate in the study were part of the Zoetis customer base and its partner distributors. Each clinic/veterinary hospital was classified as a practice that recommended heartworm chemoprophylaxis as determined by examination of purchase records that indicated acquisition of products for this purpose in the past 12 months.

Practices that met this criterion were accepted to participate in the study and were provided with a copy of the study protocol. One veterinarian in each practice was assigned to be responsible for collecting whole blood samples from clients’ dogs at that location. The number of samples to be collected at each clinic was established based on the number of cases that could be enrolled within the proposed study period, as determined by the collaborating veterinarian’s assessment of his number of medical appointments in a given period. Practices continued to collect blood samples until the targeted number of samples calculated for each region was reached.

Dogs to be included in the study were required to be more than 1 year of age and to have a history of overdue heartworm chemoprophylaxis (no oral/topical monthly products in the past 30 days or no annual injectable product in the past 365 days) or to have never received heartworm chemoprophylaxis. There were no sex or breed restrictions for inclusion. Dogs were enrolled during routine consultations or heartworm prevention awareness campaigns (called “Heartworm Days”) upon agreement of the dog owners, who were contacted by phone or social media and asked to take their dogs to the participating clinics for blood sampling. The history of overdue preventive medications was considered as motivation for direct telephone contacts. Information on mosquito density at home, travel history, outdoors time spent by the dog and perception of coughing, losing weight, lethargy or syncope were provided by the owners.

Due to the COVID-19 pandemic, “Heartworm Days” could be postponed for a week or a month to avoid mingling of clients at the practice at the discretion of the responsible veterinarian.

### Clinical and Laboratory Data

Dog owners were interviewed by the responsible veterinarian to complete a form containing information on lifestyle, demographics, health status, and clinical signs (cough, weight loss, lethargy, syncope) for an enrolled animal. Then, blood samples were collected at the clinic, transferred to blood collection tubes containing EDTA as an anticoagulant and processed by the responsible veterinarian using the Vetscan® Flex 4 Rapid Test (Zoetis; NJ USA), according to the manufacturer’s recommendations. This test uses a qualitative lateral flow immunochromatography technique that, according to the manufacturer, detects antigens of adult female *D. immitis* and antibodies against *Ehrlichia canis*, *E. ewingii*, *E. chaffeensis*, *Anaplasma phagocytophilum*, *A. platys*, and *Borrelia burgdorferi*.

### Statistical Analysis

Univariate logistic regression was used to obtain odds ratios for evaluated risk factors (breed, sex, age, weight, outdoor access, fur color and length, traveling, residence time, tick-borne antibodies, and antibiotics use) associated with the outcomes of interest and their 95% confidence intervals.

A binomial random effects logistic regression (*p* < 0.05) model was used to identify associations between each clinical sign and heartworm antigen detection. Chi-square testing was used to compare the frequency of infection within each clinical sign. Analyses were carried out in Statistical Package for the Social Sciences SPSS software (IBM R), version 25.0.

## Results

**Fig. 1:**
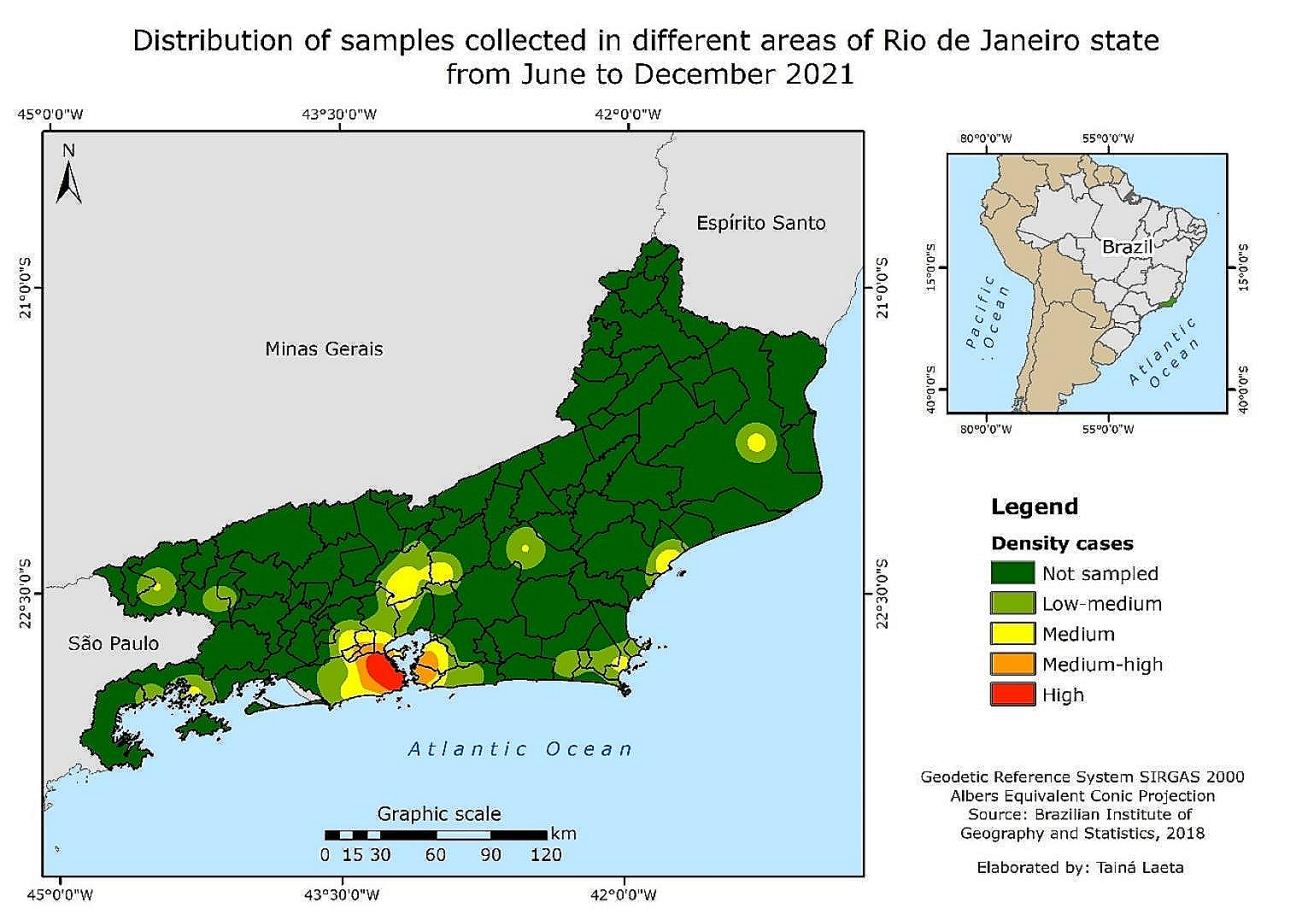
Distribution of samples collected in different areas of Rio de Janeiro state from June to December 2021

**Fig. 2:**
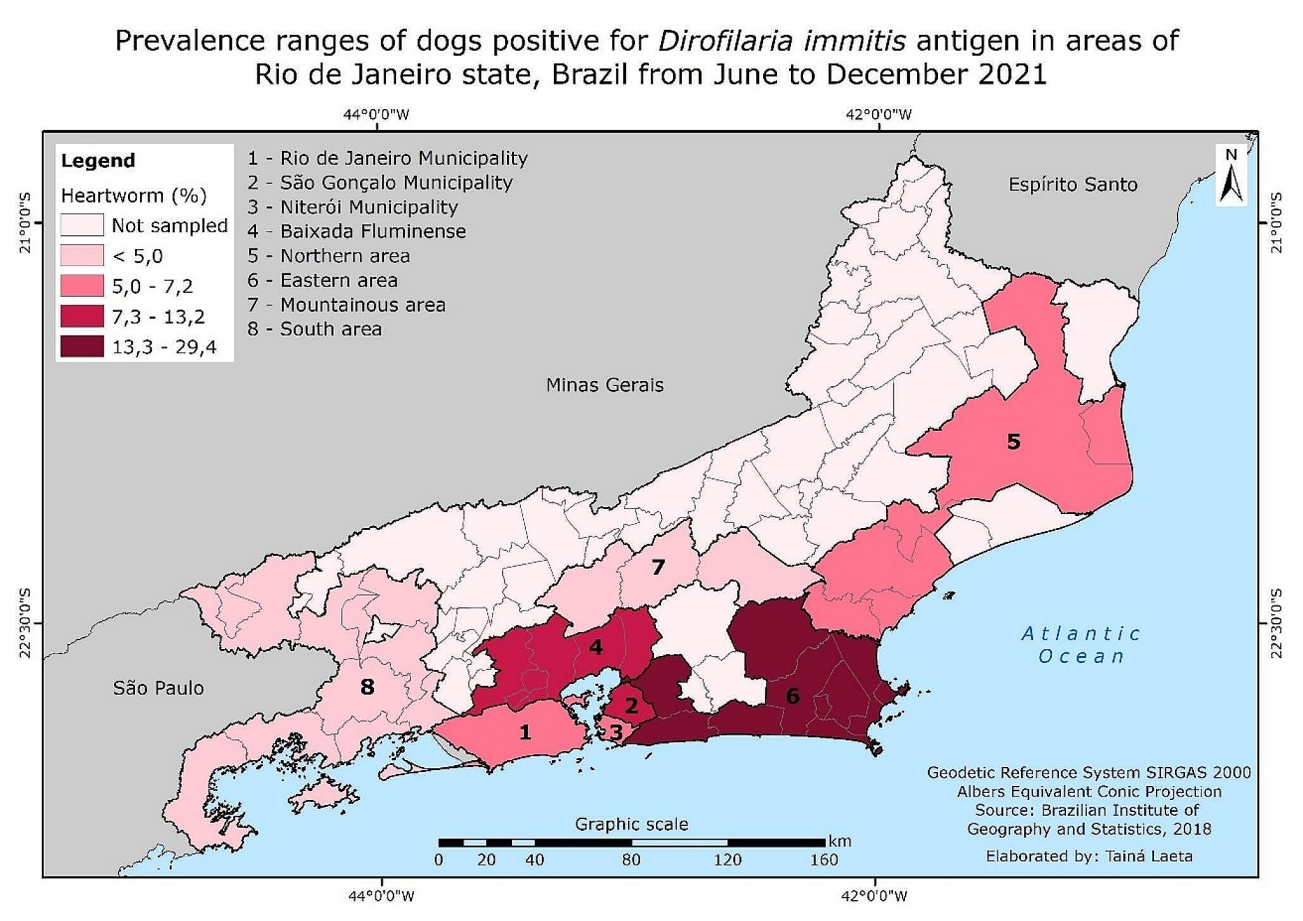
Prevalence ranges of dogs positive for *Dirofilaria immitis* antigen in areas of Rio de Janeiro state, Brazil from June to December 2021

A total of 1787 canine blood samples (107.3% of the statistically relevant sample size) were obtained from 135 practices across different areas of Rio de Janeiro state (Fig. 1), with an overall heartworm prevalence of 8.2% (146/1787). The highest heartworm prevalence was in eastern part of the state (29.4%), whereas the lowest was at mountainous area (2.4%) (Table 1; Fig. 2).

**Table 1:** Prevalence of *Dirofilaria immitis* antigen in dogs using a commercial immunochromatography technique in areas of Rio de Janeiro state, Brazil June–December 2021

Area	No. positive/No. tested	Percentage*
Rio de Janeiro municipality	32/644	5.0^a, b,d^
São Gonçalo municipality	13/104	12.5^c^
Niterói municipality	9/125	7.2^b, c,d^
Baixada Fluminense	21/159	13.2^c^
Northern area	10/149	6.7^b, c,d^
Eastern area	47/160	29.4^e^
Mountainous area	6/255	2.4^d^
Southern area	8/191	4.2^d^
TOTAL	146/1787	8.2^a, d^

*Percentages without any similar superscripts are significantly different (*p* < 0.05)

Overall, most of the participating clinics (108/135, 80%) were classified as practices that were known to recommend heartworm chemoprophylaxis to their clients (Table 2). The southern area had the lowest level of recommendation for heartworm prevention (5/17, 29.4%), and the highest level (100%) was determined for Rio de Janeiro (43/43), São Gonçalo (5/5), and Niterói (10/10) municipalities.

**Table 2:** Number of veterinary clinics that recommended heartworm chemoprophylaxis practices in their location in Rio de Janeiro state, Brazil

Area	No. clinics/Total (%)
Rio de Janeiro municipality	43/43 (100)
São Gonçalo municipality	5/5 (100)
Niterói municipality	10/10 (100)
Baixada Fluminense	8/11 (72.2)
Northern area	5/17 (29.4)
Eastern area	20/21 (95.2)
Mountainous area	9/10 (90.0)
Southern area	8/18 (44.4)
TOTAL	108/135 (80.0)

Responses were received from 71.0 to 95.4% of the dog owners in each region regarding each of the clinical signs observed for their dogs. According to the information provided by the owners, dogs positive for heartworm infection presented with cough, weight loss, lethargy, and/or syncope significantly (*p* < 0.001) for each clinical sign more frequently than in dogs negative for the disease (Table 3).

**Table 3:** Frequency of clinical signs reported by dog owners for their dogs living in Rio de Janeiro state relative to 300 *Dirofilaria immitis* antigen test results

Antigen test result	Cough	Weight loss	Lethargy	Syncope	No clinical signs
	Yes/Total (%)	Yes/Total (%)	Yes/Total (%)	Yes/Total (%)	Yes/Total (%)
Antigen positive	30/141 (21.3)	36/101 (35.6)	56/84 (66.7)	6/133 (4.5)	58/118 (49.1)
Antigen negative	207/1377 (15.0)	208/1387 (15.0)	390/1185 (32.9)	35/1572 (2.2)	945/1507 (62.7)
Total	237/1518 (15.6)	244/1488 (16.4)	446/1269 (35.1)	41/1705 (2.4)	1003/1625 (61.7)
χ^2^/*p* value	221.6/*p* < 0.001	2193.2/*p* < 0.001	1440.7/*p* < 0.001	3247.9/*p* < 0.001	8.5/ *p* < 0.005

Total numbers of results are lower than the total number of samples collected due to missing data fields in study

Only two evaluated factors were shown to enhance the risk for a dog to be infected by *D. immitis*. Short-coated dogs were at higher risk than medium- or long-coated dogs (*p* < 0.001, OR 2.62, 95% CI 1.77–3.89). Dogs presenting antibodies to tick-borne pathogens (*Ehrlichia*, *Anaplasma*, or *Borrelia*) were shown to be at higher risk of being positive for heartworm antigen (*p* < 0.001, OR 3.83, 95% CI 2.70–5.43) than dogs negative for those antibodies. Other statistically significant factors (*p* < 0.001), but not related to an increased risk of infection (OR < 1), were racial definition, age, weight, travel history, and quantity of mosquitoes observed in homes. Spending more time outdoors also did not increase the risk of a dog being infected relative to dogs that spent little time outdoors each day (Table 4).

**Table 4:** Potential risk factors for canine heartworm infection detected by Vetscan® Flex 4 Rapid Test (Zoetis) for *Dirofilaria immitis* antigen in dogs living in Rio de Janeiro state, Brazil

Risk factor	*N*	Dirofilaria immitis antigen-positive		
		*n* (%)	Odds ratio (95% CI)	*p* value
Total samples	1787	146 (8.2)		
*Breed* Yes No	933 829	58 (6.2) 85 (10.2)	0.58 (0.41–0.88)	0.0026
*Sex* Male Female	789 997	71 (9.0) 75 (7.5)	1.21 (0.87–1.71)	0.2966
*Age* ≤ 4 yr > 4 yr	755 1015	40 (5.3) 103 (10.1)	0.49 (0.34–0.72)	0.0003
*Weight* ≤ 16 kg > 16 Kg	1069 651	56 (5.2) 78 (12.0)	0.41 (0.28–0.51)	< 0.0001
*Coat color* Light Dark	430 1313	31 (7.2) 110 (8.4)	0.85 (0.56–1.28)	0.5033
*Coat length* Short Medium-long	1020 727	111 (10.9) 34 (4.7)	**2.62 (1.77–3.89)**	< 0.0001
*Long outdoor time* Yes No	1170 595	47 (4.0) 95 (16.0)	0.22 (0.15–0.32)	< 0.0001
*Travel history* Yes No	410 1342	17 (4.1) 125 (9.3)	0.42 (0.25–0.71)	0.0011
*Mosquitoes* Few Many	1184 569	57 (4.8) 89 (15.6)	0.27 (0.19–0.38)	< 0.0001
*TBD antibodies* Yes No	357 1430	66 (18.5) 80 (5.6)	**3.83 (2.70–5.43)**	< 0.0001

Total number of animals within each risk factor may be less than the number of samples collected due to the lack of completion of some fields in the study form

Data shown in bold face type designate factors that were shown to significantly increase the risk of infection with *Dirofilaria immitis*

CI, confidence interval; TBD, tick-borne disease pathogens (*Ehrlichia* spp., *Anaplasma* spp. or *Borrelia* sp.)

Nearly half of the dogs testing positive for antibodies to tick-borne pathogens (46%, 143/255) either presented no clinical signs of infection or their owners did not perceive signs of disease.

## Discussion

This is the first large survey focused on the canine population with access to veterinary services in Rio de Janeiro state, Brazil. To our knowledge, the most recent survey of heartworm prevalence completed in several areas of Rio de Janeiro state was in 2013–2014, which revealed a combined 39.7% infection rate in Mangaratiba, Niteroi, Cabo Frio, and Armação de Búzios areas [12]. This contrasts with the 8.2% prevalence rate obtained in the present study; however, samples in the present study were collected in veterinary clinics and hospitals, whereas samples in the 2014 were collected in the field and included stray and household dogs. In theory, and concluded by the authors in one survey [15], dogs with access to veterinary practices tend to have better care than dogs that lack access to veterinary care, which can justify the difference in prevalence rates between the current study and the earlier survey.

The only previous report of the prevalence among dogs receiving veterinary care in Rio de Janeiro municipality showed a prevalence rate of 7% [16], similar to findings in the present survey (5%). These numbers, when compared with the infection rate for dogs with limited access to veterinary care in the Rio de Janeiro municipality (24.6%) [17], suggest the exposure to infection experienced by local dogs is high and that owners may not fully comply with the recommendations provided by the veterinarians or that veterinarians are not regularly prescribing heartworm preventatives. However, the suggestion that veterinarians are not prescribing heartworm preventatives is reinforced by the fact that 100% of the practices in Rio de Janeiro municipality included in this study were classified as those who typically recommend heartworm chemoprophylaxis based on their recorded purchases of heartworm preventive products in the past 12 months.

In Niteroi and in the eastern region, high rates of infection (58.6% and 44.8%, respectively) were previously detected in dogs with limited access to medical care [12]. In the present study, infection rates were among the lowest in animals in Niteroi (7.2%) and the highest was in the eastern region (29.4%), noting that there appear to be high rates of heartworm chemoprophylaxis recommendation practices in both of these regions (100% and 95%, respectively). Although it is difficult to determine why the eastern area had the highest infection rate (29.4%) given the current rate of heartworm preventive products ordered by veterinarians in that region, problems with dog owners’ compliance to use preventives may have contributed to the high rates of infections observed. Conversely, the rate of infection in Niteroi, an area traditionally known for its high heartworm prevalence, was much lower (7.2%).

There is an earlier report of 20% prevalence of canine heartworm infection in the mountainous area [10]. However, at that time (1990s), heartworm preventatives were not available in Brazil, and the number of animals tested was very small (20 dogs), compared to the sample in the present study, in which a much lower prevalence was observed (2.4%, 6/255).

The prevalence found in the southern part of the state in the present survey (4.2%) shows that although there are infected dogs in the area, transmission seems to be kept at a low level, even in face of a relatively low level of veterinary practices recommending heartworm prevention (44.4%).

There is paucity of previous information about heartworm prevalence in São Gonçalo municipality, Baixada Fluminense, and the northern and southern areas of the state. Canine heartworm infection is known to be rare in these areas based on anecdotal information provided by veterinarians; therefore the relevance of 12.5%, 13.2%, 6.7%, and 4.2% infection rates respectively, may reflect a tendency of infection spreading, as it has been reported elsewhere [18]. This fact may be even more concerning because dogs in the current survey were sampled in veterinary practices and not in the field. Particularly in the southern area, only 29.4% of clinics recommend prevention, which is inconsistent with a heartworm prevalence of 4.2%. It is possible there is a hesitation by the veterinarians to prescribe heartworm preventatives because they fear being misjudged by pet owners for prescribing a drug for a disease that still has a low prevalence in the region.

In the present study, cough, weight loss, lethargy and syncope were associated with heartworm infection based on information provided by pet owners. In another recent study, cough was reported in heartworm-positive (7/11) and heartworm-negative (14/15) dogs by pet owners (consistent with rates determined by veterinarians using positive cough tracheal reflex in the study) [19]. Since dog owners in the present study reported coughing to be more frequent in heartworm-infected dogs, this clinical manifestation must be given consideration by the practitioners and should always include *D. immitis* infection as a differential diagnosis, especially when accompanied by weight loss, lethargy, and/or syncope.

Although dog owners were instructed to observe their dogs closely to be able to recognize potential signs of heartworm infection, many of them failed to report any clinical signs of the disease. Additionally, almost half of the dog owners (46%) whose dogs were antibody-positive for tick-borne pathogens failed to notice any of the classic clinical signs of those infections. Many tick-borne infections may be difficult to identify in cases when infections are asymptomatic or at least oligosymptomatic, as previously reported from a study in Brazil [20]. That study also found that observance of clinical signs routinely associated with tick-borne disease were reported for dogs negative for these pathogens. This highlights the importance of screening dogs frequently to keep updated baseline information on tick-borne exposure.

The known primary vectors of *D. immitis* in the state of Rio de Janeiro are crepuscular or nocturnal, sylvatic, hemisynantropic, and eclectic *Ochlerotatus* mosquitoes [5, 21, 22]. These vector characteristics suggest that they will feed on the easiest and most abundant host. However, they are hemisynantropic; therefore, they will tend to feed in the vicinity of a home but seldom will feed inside homes. There is no information how mosquitoes overcome long-furred hosts to penetrate the fur barriers to the skin, but the results of this survey suggest that coat length plays an important role, more important than coat color or outdoors exposure, noting that short-haired dogs were at higher risk than dogs with medium or long coats. In another study, long coat length was shown to increase the risk of infection along with white coat color [12]. In this study, coat color did not increase the risk of infection. It appears that color may be less important as an attraction factor since the insects don’t use colors as a guidance factor to find hosts.

Dogs presenting antibodies to tick-borne pathogens were at higher risk of infection by *D. immitis*. It is known that ticks are not capable of transmitting heartworm disease since the participation of mosquito species is necessary for the conversion of first-stage larvae (microfilariae) to infective third-stage larvae [14]. Therefore, the association between heartworm disease and the presence of antibodies to tick-borne pathogens may indicate that the same tick prevention failures can be observed in relation to heartworm prevention.

In conclusion, heartworm infection rates were variable (8.2% on average; 2.4– 29.4% range) in dogs with access to veterinary services across most of the areas of Rio de Janeiro state, Brazil, despite overall high availability of heartworm preventatives in the surveyed practices. This may indicate a gap in terms consistent dispensing of heartworm chemoprophylactic products by veterinarians or compliance issues for product purchase or administration by pet owners. Cough, weight loss, lethargy and syncope were the clinical signs associated with heartworm infection, and short coat and antibodies to tick-borne pathogens were the risk factors for the disease.
